# Correction: Addressing interfacial chemical corrosion in lithium metal batteries: a ferroelectric-dipole-regulation route

**DOI:** 10.1039/d6sc90161a

**Published:** 2026-08-13

**Authors:** Baolei Xu, Zeqiang Xie, Chunxiao Zhang, Liangjun Zhou, Libao Chen, Weifeng Wei

**Affiliations:** a School of Frontier Crossover Studies, Hunan University of Technology and Business Changsha 410205 China; b National Research & Development Center of Powder Metallurgy, Powder Metallurgy Research Institute, Central South University Changsha Hunan 410083 China weifengwei@csu.edu.cn

## Abstract

Correction for ‘Addressing interfacial chemical corrosion in lithium metal batteries: a ferroelectric-dipole-regulation route’ by Baolei Xu *et al.*, *Chem. Sci.*, 2026, **17**, 11744–11753, https://doi.org/10.1039/d6sc02846b.

The authors regret that they did not disclose the use of the artificial intelligence tool DeepSeek in the preparation of their article in their published Acknowledgements section. The amended Acknowledgements section is shown below.

## Acknowledgements

We would like to acknowledge financial support from the Foundation for Outstanding Research Groups of the National Natural Science Foundation of China (Grant No. 72088101), the National Natural Science Foundation of China (Grant No. 52477228, 52407257 and 52377220), and the Key Project of Xiangjiang Laboratory (No. 25XJ02001). During the preparation of this work, the authors used DeepSeek for language polishing and copy-editing. After using this tool, the authors reviewed and edited the content as needed and take full responsibility for the final publication.

The Royal Society of Chemistry apologises for these errors and any consequent inconvenience to authors and readers.

